# Comparative analysis of low- and high-dose apheresis platelets in platelet-refractory patients with hematologic malignancies

**DOI:** 10.1016/j.htct.2026.106538

**Published:** 2026-09-18

**Authors:** Suvetha Rajendran, Ratti Ram Sharma, Rekha Hans, Pankaj Malhotra, Divjot Sing Lamba

**Affiliations:** a Department of Transfusion Medicine, Post Graduate Institute of Medical Education and Research (PGIMER), Chandigarh, India; b Department of Clinical Hematology and Medical Oncology, Post Graduate Institute of Medical Education and Research (PGIMER), Chandigarh, India

**Keywords:** Platelet transfusion, Alloimmunization, Platelet refractoriness, Single donor apheresis, Crossmatch

## Abstract

**Background:**

Alloimmunization-induced platelet transfusion refractoriness represents a major clinical challenge in hematology-oncology. Optimal strategies for platelet support in this population remain to be established.

**Objective:**

This study aimed to compare the efficacy of low-dose crossmatched versus high-dose uncrossmatched single donor apheresis platelets (SDAP) in platelet-refractory patients with hematologic malignancies by assessing corrected count increment (CCI), percent platelet recovery (PPR), and post-transfusion platelet increment (PPI).

**Materials and methods:**

Forty alloimmunized, platelet-refractory patients were enrolled. Solid phase red cell adherence assay was used for crossmatching and alloimmunization detection. Patients received low-dose crossmatched SDAP (1.49 ± 0.30 × 10^11^ platelets/unit) followed by high-dose uncrossmatched SDAP (4.9 ± 1.0 × 10^11^ platelets/unit) at the subsequent transfusion. The CCI, PPR %, PPI, transfusion intervals, and World Health Organization bleeding grades were recorded and compared after each transfusion.

**Results:**

The CCI and PPR% were significantly higher following low-dose crossmatched SDAP compared to standard-dose SDAP (p-value <0.05). Compared to high-dose uncrossmatched SDAP, low-dose crossmatched SDAP produced slightly higher CCI and PPR%, but differences were not statistically significant (p-value = 0.27). PPI was significantly higher with high-dose uncrossmatched SDAP (p-value <0.01) compared with low-dose crossmatched SDAP. Transfusion intervals and bleeding episodes were similar across both groups.

**Conclusion:**

Low-dose crossmatched SDAP achieves comparable efficacy to high-dose uncrossmatched SDAP in refractory, alloimmunized patients with hematologic malignancies. These results support low-dose crossmatched SDAP as a feasible strategy to optimize transfusion support, minimize complications, and improve hemostasis management in refractory patients.

## Introduction

Platelet transfusions play a vital role in both the prophylaxis and management of bleeding in patients with hematologic malignancies [1,2]. Nevertheless, repeated exposure to allogeneic platelets can lead to platelet transfusion refractoriness, most frequently due to alloimmunization against human leukocyte antigens (HLA), which accounts for 80–90% of cases. Alloimmunization against human platelet antigens (HPA) contributes to approximately 10–20% of cases, while a subset of patients may develop antibodies against both HLA and HPA [3, 4, 5].

Platelet refractoriness occurs in 15-25% of patients with hematologic malignancies. Studies have found that even with leukocyte reduced products, 17-45% of aplastic anemia and hematology malignancy patients experience platelet refractoriness [6]. A study conducted in northern India reported around 56.7% of platelet refractoriness in multi-transfused hematological patients [7].

Refractoriness to platelet transfusion considerably compromises clinical management, increasing the risk of bleeding, resource utilization, and cost [1, 2, 3, 4, 5]. Several strategies exist to mitigate refractoriness, including transfusing HLA-matched platelets, crossmatched platelets, or escalating the platelet dose. While high-dose apheresis platelets can increase post-transfusion increments, they may also subject patients to higher donor exposures and risk of additional alloimmunization.

Previous studies have explored the prevalence and impact of platelet transfusion refractoriness and alloimmunization in patients with hematologic malignancies [7, 8, 9, 10]. However, comparative data evaluating low-dose crossmatched versus high-dose uncrossmatched SDAP specifically in the setting of confirmed alloimmunized platelet-refractory patients are limited.

Accordingly, this study was conducted to assess and compare the efficacy of low-dose crossmatched SDAP and high-dose uncrossmatched SDAP in alloimmunized, platelet-refractory patients with hematologic malignancies in terms of the corrected count increment (CCI), platelet percentage recovery (PPR%), post-transfusion platelet increment (PPI), transfusion intervals, and bleeding outcomes, so as to offer the best possible platelet product for these patients in the future.

## Materials and methods

This prospective case-control study was conducted in the Department of Transfusion Medicine, in collaboration with the Department of Clinical Haematology and Medical Oncology, at a tertiary care hospital in India between January 2023 and June 2024. The study protocol was approved by the Institutional Ethics Committee, and all patients provided written informed consent after the study objectives and procedures had been explained. The study was prospectively registered in the Clinical Trial Registry of India (CTRI/2023/06/053410).

### Patients

The inclusion criteria were age ≥18 years, no bleeding episodes at enrollment, and platelet-refractory hematology-oncology patients (diagnosed with aplastic anemia, acute myeloid leukemia [AML], lymphoma, acute lymphoblastic leukemia [ALL], or multiple myeloma) with thrombocytopenia having a corrected count increment (CCI) <5,000 at 20-24 hours post-transfusion following two consecutive single donor apheresis platelets (SDAP) transfusions. The exclusion criteria were non-refractory cases, active pyrexia, infection, use of drugs causing thrombocytopenia, splenomegaly, and bleeding episodes.

### Study design

All enrolled patients initially received a standard-dose of SDAP (3 × 10^11^ platelets/unit) and the CCI was calculated. The platelet count was measured using the Beckman-Coulter DXH 800 cell counter which works on the principle of electrical impedance. Patients with a CCI <5000 at 24 hours post-transfusion after two consecutive SDAP transfusions (receiving standard-dose of platelets) were considered platelet-refractory and included in the study. These refractory patients subsequently received a low dose, crossmatched compatible SDAP unit (1.5 × 10^11^ platelets/unit). The PPI, CCI and PPR were recorded 24 hours post-transfusion. Patients were followed for bleeding manifestations, and the interval between two requisitions was noted. For the next transfusion episode, the same patient was administered a high-dose, uncrossmatched SDAP (≥4.5 × 10^11^ platelets/unit). Thus, each patient served as their own control (standard dose) and case (low-dose/high-dose) at different time points. Again, the patient was followed for bleeding episodes with the next requisition for SDAP platelets and interval between the two requisitions being noted.

The following calculations were used:

$$\begin{array}{c} Corrected\;Count\;Interval\;\left(CCI\right)=\\ \frac{\left(post-transfusion\;platelet\;count-pre-transfusion\;platelet\;count\right)\times BSA}{platelet\;transfused\;\left(\times 10^{11}\right)} \end{array}$$

$$\begin{array}{c} Percent\;Platelet\;Recovery\;\left(PPR\right)=\\ \frac{\left(post-transfusion\;platelet\;count-pre-transfusion\;platelet\;count\right)\times TBV\times 100}{platelet\;transfused\;\left(\times 10^{11}\right)} \end{array}$$

$$\begin{array}{c} Post-transfusion\;Platelet\;Increment\left(PPI\right)=\\ post\;transfusion\;platelet\;count-pre\;transfusion\;platelet\;count\;\left(/L\right) \end{array}$$

### Platelet collection and processing

ABO identical SDAPs, obtained from replacement or voluntary donors who fulfilled the criteria for apheresis platelet donation as per the Directorate General of Health Services (DGHS) technical manual, were prepared using an automated cell separator (TRIMA Accel, Terumo BCT, Lakewood, Colorado, USA or AMICUS, Fresenius Kabi, Germany) [8]. Written informed consent was obtained from all donors prior to the procedure.

To establish the required transfusion doses, apheresis platelet units were customized as follows: standard-dose units contained 3 × 10^11^ platelets, whereas low-dose units were produced either by splitting a standard-dose unit into two equal aliquots (1.5 × 10^11^ platelets/unit) using a Terumo sterile connecting device or by dividing a double-dose product (6 × 10^11^) into a low-dose aliquot (1.5 × 10^11^ platelets/unit) and a high-dose aliquot (≥4.5 × 10^11^ platelets/unit)

### Platelet crossmatching procedure

Platelet crossmatching was performed using the CAPTURE-P solid phase system on an automated immunohematology analyzer (Galileo, Immucor, Inc, Norcross, GA USA). For crossmatching, ABO-matched SDAP units from the daily inventory were selected and tested against patient sera.

### Statistical analysis

Data were coded and recorded in MS Excel and analyzed using SPSS v23 (IBM Corp.). Descriptive statistics are presented as means ± standard deviation (SD) or medians and interquartile ranges for continuous variables, and as frequencies and percentages for categorical variables. Normality for continuous data was assessed with the Shapiro-Wilk test. As each patient received all three interventions, paired t-tests were used for intra-individual comparisons: standard-dose versus low dose, standard-dose versus high dose, and low-dose versus high dose. A p-value <0.05 was considered statistically significant.

## Results

A total of 117 patients with hematologic malignancies received standard-dose SDAP and were screened for platelet refractoriness (CCI <5000 at 24 hrs.). Among them,70 patients (59.8%) were refractory with a mean CCI of 2955 ± 1839 and mean PPR of 10 ± 8.5%. Of these 70 refractory patients, 40 (57%) patients received low-dose crossmatched compatible SDAP units while 39 (56%) were given high-dose uncrossmatched SDAP units and were followed up for platelet response indicators and clinical outcomes. Thirty refractory patients were excluded from the intervention analysis for the following reasons: absence of alloimmunization (n = 9), unascertained alloimmunization status (n = 8), sampling errors (n = 5), loss to follow-up (n = 3), bleeding episodes requiring standard-dose platelet transfusion (n = 2), and lack of subsequent SDAP requisitions (n = 3). Patients with non-immunological causes of platelet refractoriness were excluded. Among patients with suspected immune-mediated refractoriness, nine patients (not alloimmunized) tested negative for alloimmunization by a solid phase red cell adherence assay, likely due to assay limitations, including the limited donor pool (13 donors) used in the Neo Immucor Capture-P Ready-Screen equipment, so these patients were not included in the study either. Hence, collectively 40 patients received low-dose SDAP units and of those 40, only 39 patients received high-dose SDAP units.

Of the 70 platelet refractory patients, 39 were males and 31 were females (male: female = 1.2:1: Table 1). The underlying diagnoses in this cohort included ALL (n = 18; 25.7%), AML (n = 17; 24.3%), aplastic anemia (n = 14; 20%), acute promyelocytic leukemia (APML - n = 11; 15.7%), myelodysplastic syndrome (MDS - n = 3; 4.2%), multiple myeloma (n = 2; 3%), and other conditions such as Hodgkin’s lymphoma, myelofibrosis, and chronic myeloid leukemia (CML - n = 5; 7.1%).

**Table 1:** Demographic characteristics of the study population (n = 70).

Variable	Mean ± Standard deviation
Age (years)	40.6 ± 16.4
Sex (Male: Female)	1.2:1
Weight (kg)	64.5 ± 8.8
Height (cm)	166.2 ± 8.8
Body Surface Area (m^2^)	1.5 ± 0.1
Blood Volume (mL)	4244.8 ± 815.6
No. of previous platelet transfusions	11.2 ± 5.7

### Comparative assessment of post transfusion recovery profiles in different platelet dose groups

A. Standard-dose versus low-dose (crossmatch) single donor apheresis platelets

The mean pre-transfusion platelet counts did not differ significantly between patients who received standard-dose and low-dose crossmatched SDAP (p = 0.21). Similarly, no statistical difference was observed in the PPI or transfusion interval between the two groups (p = 0.93 and p = 0.19, respectively). In contrast, both the CCI and PPR% demonstrated statistically significant improvement in the low-dose crossmatched group compared with the standard-dose uncrossmatched group (p <0.01 for both - Table 2). However, one WHO grade 1 bleeding episode was observed in a patient who was transfused with low-dose crossmatched SDAP; this patient received a standard-dose SDAP for the subsequent requisition, and was consequently withdrawn from the study. So, from this group of forty patients who received low-dose crossmatched SDAP, only 39 patients were given high-dose uncrossmatched SDAP units.

B. Standard-dose versus high-dose (uncrossmatched) single donor apheresis platelets

**Table 2:** Comparison of post transfusion platelet response indicators for standard-dose versus low-dose crossmatched platelets in refractory patients.

Parameter (Mean ± SD)	Standard-dose (3.1 ± 0.14 × 10¹¹ platelets/unit) (n = 40)	Low-dose (1.49 ± 0.30 × 10¹¹ platelets/unit (n = 40)	p-value
Pre-transfusion platelet count (x 10^3^/µL)	11.7 ± 7.1	9.9 ± 7.3	0.21
Post transfusion platelet count (x 10^3^/µL)	18.3 ± 8.1	16.7 ± 10	0.42
Post-transfusion platelet increment (× 10^3^/µL)	6.5 ± 3.8	7 ± 5.5	0.93
CCI	2905 ± 1613.7	5180 ± 4356.5	<0.01
PPR (%)	10.1 ± 7.7	18 ± 12.4	<0.01
Interval to next transfusion (days)	1.3 ± 0.5	1.5 ± 0.6	0.19
Bleeding episode	No bleeding episode	(Grade 1 bleeding)	-

CCI: Corrected Count Increment; PPR: Percentage Platelet Recovery

The pre-transfusion platelet count in the standard-dose uncrossmatched SDAP group and high-dose uncrossmatched SDAP group were not significantly different (p = 0.30). A total of 39 patients were subsequently transfused with high-dose uncrossmatched SDAP units, following initial transfusion with low-dose crossmatched SDAP, and were evaluated for post-transfusion parameters. The mean PPI after standard-dose SDAP was 6.5 ± 3.8 × 10^3^/µL which was significantly lower than the mean PPI of 16.0 ± 7.5 × 10³/µL achieved with high-dose uncrossmatched SDAP (p <0.01). The CCI and PPR% were significantly higher with high-dose uncrossmatched SDAP units (p <0.05 for both). In contrast, the transfusion interval did not differ significantly between the two groups (p = 0.68 - Table 3).

C. Low-dose (crossmatch) SDAP versus high-dose (uncrossmatched) single donor apheresis platelets

**Table 3:** Comparison of post transfusion platelet response indicators in standard-dose versus high-dose uncrossmatched platelet in platelet refractory patients.

Parameter (Mean ± SD)	Standard-dose (3.1 ± 0.14 × 10¹¹platelets/unit) (n = 40)	High-dose (4.9 ± 1.0 × 10¹¹ platelets/unit) (n = 39)	p-value
Pre-transfusion platelet count (x 10^3^/µL)	11.7 ± 7.1	10.3 ± 5.2	0.30
Post transfusion platelet count (x 10^3^/µL)	18.3 ± 8.1	26.3 ± 9.6	<0.05
Post transfusion platelet increment (× 10^3^/µL)	6.5 ± 3.8	16.0 ± 7.5	<0.01
CCI	2905 ± 1613.7	3947.3 ± 2293.3	<0.05
PPR (%)	10.1 ± 7.7	13.8 ± 7.4	<0.05
Interval to next transfusion (days)	1.3 ± 0.5	1.5 ± 1.1	0.68
Bleeding episode	No bleeding episode	No bleeding episode	

CCI-Corrected Count Increment; PPR-Percentage Platelet Recovery

The mean pre-transfusion platelet count did not differ significantly between patients receiving low-dose crossmatched SDAP and those receiving high-dose uncrossmatched SDAP (p = 0.79). However, the PPI was significantly higher with high-dose uncrossmatched SDAP transfusions (p <0.01). Although both the CCI and the PPR% were higher with low-dose crossmatched SDAP, the differences were not statistically significant (p = 0.27). The mean transfusion interval was comparable between the two groups, with 1.55 ± 1.12 days for high-dose uncrossmatched SDAP and 1.37 ± 0.59 days for low-dose crossmatched SDAP (p = 0.59). Among the 40 patients refractory to platelet transfusion after receiving low-dose crossmatched SDAP, one patient did not require a further transfusion as the platelet count improved with the first unit; therefore, only 39 patients subsequently received high-dose uncrossmatched SDAP (Table 4).

**Table 4:** Comparison of post transfusion platelet response indicators in low-dose crossmatched versus high-dose uncrossmatched platelet in platelet refractory patients.

Parameter (Mean ± SD)	Low-dose (1.49 ± 0.30 × 10¹¹ platelets/unit) (n = 40)	High-dose (4.9 ± 1.0 × 10¹¹ platelets/unit) (n = 39)	p-value
Pre-transfusion platelet count (x 10^3^/µL)	9.9 ± 7.3	10.3 ± 5.2	0.79
Post transfusion platelet count (x 10^3^/µL)	16.7 ± 10	26.3 ± 9.6	<0.05
Post transfusion platelet increment (× 10^3^/µL)	7 ± 5.5	16 ± 7.5	<0.01
CCI	5180 ± 4356.5	3947.3 ± 2293.3	0.27
PPR (%)	18 ± 12.4	13.8 ± 7.4	0.27
Interval to next transfusion (days)	1.3 ± 0.5	1.5 ± 1.1	0.59
Bleeding episode	1 (grade 1 bleeding)	No bleeding episode	

CCI-Corrected Count Increment; PPR-Percentage Platelet Recovery

### Trends in post transfusion response indicators

Post-transfusion response indicators were assessed using the CCI in platelet refractory patients after transfusion with low-dose crossmatched and high-dose uncrossmatched SDAP. An expected CCI of ≥5000 was observed in 45% of the patients following low-dose crossmatched SDAP transfusion, whereas only 25% of the patients met this criterion after receiving high-dose uncrossmatched SDAP. When evaluating the PPR% as a post-transfusion response indicator in these refractory cases, an expected PPR% of ≥30% was found in 20% of patients after low-dose crossmatched SDAP transfusion, and none of the patients achieved a PPR% ≥30% after a transfusion with high-dose uncrossmatched SDAP units (Table 5).

**Table 5:** CCI and PPR % response after transfusion of low-dose crossmatched SDAP and high-dose uncrossmatched SDAP in platelet refractory population.

Study group	CCI assessed	Less than Expected CCI	Expected CCI	PPR% assessed	Less than Expected PPR%	Expected PPR%
Low-dose crossmatched SDAP	n = 40	<5000 (55%) (n = 22)	≥5000 (45%) (n = 18)	n = 40	<30 (80%) (n = 32)	≥30% (20%) (n = 8)
High-dose uncrossmatched SDAP	n = 40	<5000 (75%) (n = 29)	≥5000 (25%) (n = 10)	n = 40	<30 (100%) (n = 39)	≥30% (0%) (n = 0)

CCI: Corrected Count Increment; PPR: Percentage Platelet Recovery

## Discussion

This study was conducted to evaluate the efficacy of low-dose crossmatched and high-dose uncrossmatched SDAP in platelet transfusion-refractory patients with hematologic malignancies. Platelet transfusion refractoriness was observed in 59.8% (70 out of 117) of patients, of whom 40 (57.1%) were alloimmunized. This rate of alloimmunization is lower than that reported in earlier studies from the same institute. In a study by Chavan et al., refractoriness was observed in 55.8% of patients with hematologic malignancies, with 72% showing alloimmunization [9]. In comparison, the present cohort had fewer alloimmunized patients and a higher proportion of males. Similarly, Kumawat et al. reported in a study on multi-transfused patients with hematologic malignancies that 23.2% (7 out of 30) were refractory at baseline, while 83.33% (25 out of 30) developed alloimmunization during the course of the study, which was also higher than in the current cohort [7]. This difference may be attributed to the early adoption of SDAP transfusion in this study, minimizing multiple donor exposures associated with random donor platelet transfusions. The alloimmunization rate in this study was also lower than that reported by Mangwana et al. [10], but was comparable to the findings of Bajpai et al. [11], who reported alloimmunization in 60% of patients. A higher incidence of alloimmunization was observed among patients with a history of pregnancy and previous transfusions.

In the present study, the age of patients ranged from 18-70 years, with the majority of refractory cases (n = 30; 42.9%) between 18 and 30 years. In contrast, Kumawat et al. [7] reported a median age of 45 years, while Mangwana et al. [10] observed that most patients were 60–70 years old (31.3%), with body surface area and total blood volume values comparable to the current cohort. The difference may be due to inclusion of solid organ malignancies along with hematological cases in their study. Bajpai et al. [11] reported an age range of 13–75 years with a mean of 34.9 (± 17.8) years, which was comparable to the findings of this study.

### Studies on different doses of platelets

Various studies have been conducted to determine the efficacy of different doses of platelets and crossmatched platelets in a variety of patient populations, including patients with hematologic malignancies and multi-transfused patients with solid tumors. In this study, multi-transfused patients with hematologic malignancies were initially transfused with a standard dose of SDAP and assessed for refractoriness based on the 24-hour CCI. Refractory patients were subsequently transfused with low-dose crossmatched SDAP units at their next requisition, and the response was evaluated in terms of CCI and PPR%. These parameters were then compared with those obtained following transfusion with standard-dose SDAP. It was observed that CCI and PPR% were comparatively better (p <0.01 for both) with low-dose crossmatched SDAP units.

Following transfusion of the low-dose crossmatched units, the same patients received high-dose uncrossmatched SDAP at their next platelet request, and CCI and PPR% were again assessed. When compared with values obtained after a transfusion of low-dose crossmatched SDAP, the mean CCI and PPR% were higher with low-dose crossmatched units compared with high-dose uncrossmatched SDAP, though the differences were not statistically significant (p = 0.27 for both).

A study conducted by Dhiman et al. [12] at our center compared different SDAP doses (standard-dose of 3.1 ± 0.14 × 10¹¹ platelets/unit versus a low-dose of uncrossmatched SDAP 1.49 ± 0.30 × 10¹¹ platelets/unit versus a high-dose of 4.9 ± 1.0 × 10¹¹ platelets/unit) in non-refractory cases. The authors observed no statistically significant differences between standard-dose and low-dose SDAP in terms of CCI, PPR%, and transfusion interval (p = 0.92, p = 0.89, and p = 0.85, respectively). However, when CCI and PPR% were compared between low-dose and high-dose SDAP, statistically significant differences were seen (P = 0.04 and P = 0.05), favoring low-dose units, although the transfusion interval did not differ significantly (P = 0.21).

In the present study, which focused on platelet-refractory patients with hematologic malignancies, the CCI and PPR% were significantly better with low-dose crossmatched SDAP compared to standard-dose SDAP (p <0.01 for both). Furthermore, both post-transfusion indicators were better with low-dose crossmatched units compared to high-dose uncrossmatched SDAP, albeit without statistically significant differences (p = 0.26 and p = 0.59, respectively). Consequently, low-dose crossmatched platelets emerged as a superior choice for SDAP transfusion in refractory alloimmunized patients, although transfusion intervals remained comparable, consistent with findings by Dhiman et al. [12] with uncrossmatched low-dose SDAP.

In a randomized controlled trial conducted by Slichter et al. [13], the definitions of low and standard doses differed from the present study, though their high dose was similar. Patients received prophylactic platelet transfusions with low, medium, and high doses (1.1 × 10¹¹, 2.2 × 10¹¹, or 4.4 × 10¹¹ platelets/m² body surface area, respectively) when their platelet count was ≤1 × 10^9^/L. These doses were broadly comparable to those used in this study. A total of 1272 patients were evaluated for the primary endpoint after one transfusion, with response rates of 71%, 69%, and 70% for low, medium, and high doses, respectively, without statistically significant differences. The authors concluded that low and high doses had no impact on bleeding incidence. In contrast, the current study compared low-dose crossmatched and high-dose uncrossmatched SDAP in the same refractory patients and observed that, with standard-dose SDAP, two patients (4.2%) experienced bleeding (one grade 1 and one grade 2). Similarly, one patient (2.5%) had grade 1 bleeding after low-dose crossmatched SDAP and was subsequently excluded from the study and managed with standard-dose SDAP. Thus, bleeding outcomes in this study did not differ significantly across standard-dose, low-dose crossmatched, and high-dose uncrossmatched SDAP transfusions.

Another randomized controlled trial by Heddle et al. [14] compared standard and low-dose platelet transfusion strategies (SToP) in thrombocytopenic patients. In that study, low-dose platelets were defined as 1.5-3.0 × 10¹¹ platelets/unit, and standard-dose as 3.0-6.0 × 10¹¹ platelets/unit, with the primary endpoint being grade 2 or higher bleeding. Three grade 4 bleeding episodes occurred in patients receiving low-dose platelets, compared with none in the standard-dose group. The doses differed from the present study in terms of definitions: low-dose SDAP was 1.5 × 10¹¹ platelets/unit, standard-dose was 3.0 × 10¹¹ platelets/unit, and high-dose was 4.9 ± 1.0 × 10¹¹ platelets/unit. Additionally, all three doses were compared in the same refractory, alloimmunized patients across subsequent transfusion episodes.

### Studies on crossmatched platelets in refractory patients

A previous study at this institute by Chavan et al. [9] compared standard-dose crossmatched SDAP (3.1 ± 0.14 × 10¹¹ platelets/unit) with standard-dose uncrossmatched SDAP in platelet-refractory patients with hematologic malignancies (n = 38). Post-transfusion parameters (CCI, PPR%, and PPI) were significantly better after the crossmatched SDAP transfusion (p <0.01), leading the authors to conclude that crossmatched SDAP is superior to uncrossmatched SDAP in refractory patients.

Similarly, this study included platelet-refractory patients with hematologic malignancies, but low-dose crossmatched SDAP units were transfused followed by high-dose uncrossmatched SDAP units in the same group of patients. This allowed an assessment of post-transfusion response indicators (CCI, PPR%, and PPI) not only by crossmatch status but also by dose. When comparing standard-dose SDAP with low-dose crossmatched SDAP, the latter showed statistically significant improvements in CCI and PPR% (p <0.01), though PPI did not differ significantly (p = 0.93). On comparing low-dose crossmatched SDAP with high-dose uncrossmatched SDAP, the CCI and PPR% were again better with low-dose crossmatched units, albeit without statistical significance (p = 0.26 for both). These findings were consistent across AML and ALL patients. Post-transfusion platelet increments, however, were higher with high-dose uncrossmatched SDAP, which followed standard-dose and low-dose transfusions sequentially in the study design. Overall, the findings reinforce that crossmatched SDAP units are superior, and that splitting a standard-dose of crossmatched SDAP into two low-dose units for the same patient can reduce donor exposure while ensuring availability for refractory cases.

### Trends in post-transfusion response indicators using different single donor apheresis platelet doses in refractory patients

In the study by Dhiman et al. [12] on different platelet doses, 57.1% of participants achieved an expected CCI ≥10,000 with standard-dose SDAP, 66.6% achieved CCI ≥5,000 with low-dose SDAP, and none achieved CCI ≥20,000 with high-dose SDAP. In this study, focusing exclusively on refractory patients, an expected CCI ≥5,000 was used as the benchmark. Among those who received low-dose crossmatched SDAP, 45% achieved this target, whereas only 25% achieved it with high-dose uncrossmatched SDAP. This difference likely reflects the benefit of crossmatching in refractory patients, regardless of dose. Positive correlations were also observed of CCI and PPR% with both body surface area and total blood volume, consistent with the known proportionality of these indicators to patient size.

## Limitations

Although alloimmunized patients were included in the study, antibody specificity was not further characterized. The Neo Immucor Capture-P Ready-Screen assay employs test wells coated with platelets from 13 different donors and is designed to determine the presence or absence of platelet alloimmunization rather than to identify the specific antibody involved. Consequently, while the screening panel detects alloimmunization related to both HPA and HLA antibodies, it does not allow differentiation between them. Therefore, separate reporting of HPA- and HLA-specific alloimmunization rates was beyond the scope of this study. As this study was time-bound and conducted at a tertiary care center, certain limitations affected patient follow-up, particularly among those with terminal illnesses. Additionally, technical errors encountered with the equipment resulted in only 40 of the 70 initially recruited patients being effectively enrolled in the study. So, further studies with a larger sample size are required for validation.

## Conclusion

From the present study, it was concluded that low-dose crossmatched SDAP is a better alternative to standard-dose or high-dose uncrossmatched SDAP in refractory alloimmunized patients with hematologic malignancies. When a standard or high-dose SDAP product is crossmatch-compatible, it can be divided into two or three low-dose units, thereby minimizing donor exposure and preserving crossmatched units for future transfusions. Since low-dose SDAP units are transfused after crossmatching, they yield better post-transfusion response indicators than uncrossmatched high- or standard-dose units in refractory alloimmunized patients. Given that these patients have already developed antibodies to platelet antigens, crossmatched platelets should be preferred irrespective of dose.

Thus, platelet crossmatching represents a cost-effective alternative to HLA matching, improving transfusion outcomes and reducing the risk of further alloimmunization. Transfusion of low-dose crossmatch-compatible platelets may therefore be an effective management strategy in resource-constrained settings like India. Overall, low-dose crossmatched SDAP improves patient outcomes by reducing platelet dose per transfusion episode, minimizing donor exposure, and lowering overall treatment costs.

## Declaration of generative AI in scientific writing

During the preparation of this work the author(s) used CHAT GPT for Language Editing. After using this tool/service, the author(s) reviewed and edited the content as needed and take(s) full responsibility for the content of the publication.

## Ethics approval statement

The study design was approved from the institutional ethics committee.

## Patient consent statement

An Informed written consent was obtained at the time of enrolment of patient in the study

## Clinical trial registration

The research was registered with clinical trial registry number - CTRI/2023/06/053410.

## Conflicts of interest

Nil.

## Data Availability

The data that support the findings of this study are available from Dr Suvetha Rajendran (First author) upon reasonable request.
